# Artificial Intelligence-based predictive models for adverse blood donor reactions: a systematic review of immediate and delayed events and clinical data approaches

**DOI:** 10.1186/s12911-026-03584-0

**Published:** 2026-05-25

**Authors:** Mahdie ShojaeiBaghini, Mohammad Mehdi Ghaemi, Alihasan Ahmadipour

**Affiliations:** 1 Medical Informatics Research Center, Institute for Futures Studies in Health, Kerman University of Medical Sciences, Kerman, Iran; 2 Modeling in Health Research Center, Institute for Futures Studies in Health, Kerman University of Medical Sciences, Kerman, Iran; 3 Social Determinants of Health Research Center, Yasuj University of Medical Sciences, Yasuj, Iran

**Keywords:** Adverse donor reactions (ADRs), Donor safety, Artificial intelligence (AI), Newcastle-Ottawa Scale (NOS) tool

## Abstract

**Background:**

A significant challenge in blood donation is the occurrence of adverse donor reactions (ADRs) and their subsequent negative impact on the blood supply and public health. A promising strategy to mitigate these events is the deployment of non-invasive, cost-effective artificial intelligence (AI) models for donor screening and monitoring. This study aims to systematically review the AI models utilized in the identification and prediction of ADRs.

**Methods:**

This study used a systematic review approach in line with the PRISMA 2020 guidelines. A search was performed across various databases, including Web of Science, PubMed, Embase, Google Scholar, and Scopus. The results were combined narratively and presented using descriptive statistics. The quality of eligible studies was assessed using the Newcastle-Ottawa Scale (NOS) tool.

**Results:**

Among the 13 studies, nine were classified as immediate reactions and four as delayed reactions. The commonly used models included regression models, classical statistical models, and machine learning algorithms such as Random Forests, Gradient Boosting Machines (GBMs), XGBoost, and Artificial Neural Networks (ANNs). The main standard evaluation metrics for the models included Odds Ratio, Precision-Recall Area Under the Curve (PR-AUC), F1 Score, Precision, and Recall.

**Conclusions:**

Adverse reactions among blood donors negatively impact donor retention and, by extension, the stability of the blood supply for patient care. In this context, AI models may offer a promising tool for supporting the prediction and monitoring of ADRs. However, the available studies are not sufficient to support the widespread adoption of these models in clinical or operational decision-making. Heterogeneity in study design, outcomes, and evaluation metrics together with limitations such as limited implementation, risk of bias, unclear reference standards, and a lack of external validation has constrained the interpretability and generalizability of the findings. Therefore, future research with more rigorous study designs, standardized reporting, harmonized evaluations, and external validation is essential to establish the effectiveness and reliability of these models.

**Supplementary Information:**

The online version contains supplementary material available at 10.1186/s12911-026-03584-0.

## Introduction

### Background and overview of ADRs

Blood donation is a crucial intervention in the healthcare system, involving the voluntary collection of blood from low-risk donors for storage and subsequent transfusion [1]. Globally, more than 100 million units of blood are donated annually, providing lifesaving treatment for millions of people [2]. Adverse reactions associated with blood donation and transfusion are broadly categorized into adverse donor reactions (ADRs) [3] and adverse transfusion reactions (ATRs) [4, 5]; the former affects donors, whereas the latter impacts recipients. ADRs encompass a spectrum of signs and symptoms of varying severity experienced by the donor, which may be self-reported or clinically observed by medical staff [6]. These adverse events can manifest before, during, or after the donation process [7]. ADRs are primarily classified into immediate and delayed reactions. Immediate reactions occur acutely at the donation site or shortly thereafter; they are generally transient and clinically manageable. In contrast, delayed reactions emerge after the donor has left the facility, or develop cumulatively following repeated donations, thereby presenting greater management challenges and often necessitating longer-term follow-up [8].

The most frequently reported delayed ADRs include bruising (30.9%), arm pain (26.2%), and general weakness (18.9%), with the vast majority of these cases (98.89%) occurring outside blood donation centers [7]. Among the most prevalent acute ADRs are vasovagal reactions (VVRs), which have an incidence rate ranging from 0.45% to 0.66% across various populations [9]. Characterized by a sudden drop in heart rate and/or blood pressure triggered by stress-inducing factors, VVRs are also a recognized complication in interventional pain management procedures [10].

Globally, the reported incidence of ADRs varies significantly across different regions and demographics. General estimates indicate an incidence rate of approximately 0.20%, with higher frequencies consistently observed among first-time donors and younger individuals aged 18 to 29 years [11]. In India’s National Monitoring Program, the overall incidence rate of adverse reactions was 2.45 per 1,000 donations, with general reactions being the most common (83.7%) [12]. In a German study, 27.5% of donors experienced at least one reaction, with the majority (64.4%) occurring after leaving the blood donation center [13]. Furthermore, Iranian data highlight a discrepancy between actual and reported rates, estimating a true ADR incidence of 2%, primarily localized reactions compared to an officially reported rate of just 0.5% [14].

Evidence consistently demonstrates that adverse reactions severely compromise blood donor retention rates [15]. For instance, among platelet apheresis donors, immediate adverse reactions affected approximately 7.6% of donors and significantly reduced the donor return rate, particularly among women, those under 30 years of age, and first-time donors [16]. Preventive strategies for VVRs vary. These techniques include water loading [17], active muscle tension [18], social support, distraction, and even the administration of pharmacological interventions such as low-dose benzodiazepines [19]. Although these preventive measures may be effective, they can be labor-intensive. Therefore, there is a pressing need to develop innovative and efficient preventive methods for the timely identification, prediction, and prevention of such reactions.

### Innovative approaches in predicting and preventing ADRs

One innovative preventive method is the use of non-invasive, low-cost Artificial Intelligence (AI) models to monitor donors and identify and predict adverse reactions, including VVRs. Recent research has demonstrated promising applications of AI in predicting reactions in blood donation environments. Rudokaite and colleagues developed innovative methods using facial microexpressions and thermal imaging, achieving F1 scores of 0.82 and 0.86 by analyzing donors’ involuntary facial reactions in the waiting room before donation. Their facial temperature analysis showed excellent performance, with a sensitivity of 0.87 and a specificity of 0.84, indicating that temperature changes in the area beneath the nose, chin, and forehead were the most significant predictive features [20]. Zhao and colleagues developed a nomogram to predict VVRs in plasma donation using data from 120,448 donors, achieving a C-index of 0.916. Their model revealed that gender, season, donor status, weight, heart rate, donation duration, and donation cycle were independent risk factors for VVR [9]. Studies on ADRs have been sparse and heterogeneous, highlighting the need for a systematic review to synthesize and analyze the existing evidence.

### Aim of study and methodology

This systematic review aims to elucidate diagnostic and preventive strategies by evaluating existing models, assessment criteria, reaction typologies, blood donation modalities, and risk factors contributing to adverse reactions. The findings are intended to guide future research and empower transfusion medicine specialists to anticipate adverse events, implement targeted management protocols, ensure donor retention, and ultimately increase blood donation rates.

While certain outcomes analyzed may not manifest directly as acute ADRs, they serve as critical indicators of long-term complications or indirect effects associated with the donation process, thereby impacting overall donor health. Consequently, the primary objective of this review is to systematically identify and critically evaluate AI models designed to predict both immediate and delayed ADRs. This encompasses an assessment of methodological quality and clinical readiness, including the categorization of input data and algorithmic architectures (ranging from standard regression models to deep learning networks), alongside a synthesis of performance metrics and validation methodologies.

Furthermore, the Newcastle-Ottawa Scale (NOS) was employed to evaluate study quality and risk of bias. Finally, we discuss the challenges and prerequisites for integrating these predictive models into clinical workflows, such as electronic health records (EHRs), while addressing pertinent ethical and legal considerations. The specific study objectives were formulated in accordance with the Population, Intervention/Exposure, Comparator, Outcome (PICO/PECO) framework. The population (P) consists of blood donors, while the intervention/exposure (I/E) refers to the design or validation of AI models to predict ADRs. This review does not include a specific comparator (C) group. The intended outcome (O) is the ability of these AI models to identify and predict ADRs.

## Method

### Study methodology

#### Study design

This review was conducted according to the guidelines of the Preferred Reporting Items for Systematic Reviews and Meta-Analyses (PRISMA, 2020) [21]. Additionally, the study followed the protocol registered in PROSPERO with the identifier CRD420251164704.

#### Inclusion and exclusion criteria

The inclusion criteria consist of English-language studies that focus on the application of AI in the identification and prediction of immediate and delayed ADRs, with both the abstract and full text accessible. Exclusion criteria include studies for which the full text is unavailable, those unrelated to the review topic, or those published in languages other than English. Non-research articles such as reviews, letters, and book chapters were also excluded. Furthermore, studies that were not peer-reviewed or were presented at conference proceedings were excluded.

#### Information sources

This systematic review utilized the following databases as primary information sources: Web of Science, PubMed, Embase, Google Scholar, and Scopus. The database searches were conducted for studies published before October 5, 2025.

#### Search strategy

The search strategy for this systematic review was formulated to comprehensively identify literature pertaining to AI and ADRs. The approach incorporated keywords aligned with the study’s objectives, such as “Adverse Donor Reactions”, “Blood Donors”, and “Artificial Intelligence”, and combined using Boolean operators (AND, OR). To refine the search, Medical Subject Headings (MeSH) terms were applied within the PubMed database. The search was executed by a single author. Furthermore, the reference lists of the retrieved articles were not manually screened for additional studies. The detailed search strategy for each database is provided in Table A1 of the Appendix.

#### Study selection

Based on the identified studies, key information, including titles, abstracts, author names, institutional affiliations, journal titles, and publication years, was collected. This information was then entered into EndNote Reference Management Software (version 2021) to facilitate data extraction and organization. After removing duplicates, two authors independently reviewed the titles and abstracts. The Inter-rater reliability between the two reviewers during the title and abstract screening phase was high (Cohen’s Kappa = 0.85). Discrepancies were resolved through discussion, and if necessary, consultation with a third author was sought. Subsequently, full-text articles of the relevant studies were retrieved. Studies that did not meet the inclusion criteria after full-text review were excluded, and the reasons for exclusion are provided in the Preferred Reporting Items for Systematic Reviews and Meta-Analyses (PRISMA) flowchart.

### Data collection and analysis

#### Data items

After final study selection, two authors independently extracted key data using a data extraction form in Microsoft Word 2021. The third author resolved any disagreements regarding the data. The extracted data included the study identification number, first author, year, study type, country, study objective, target population, type of donated product, type of donor adverse reactions, Immediate/Delayed classification, AI algorithms or models, model evaluation criteria, data type, and main findings. The results of the data extraction process are presented in Tables A2 and A3 in the Appendix.

#### Quality and risk of bias assessment

In this study, the NOS tool was used to assess quality and risk of bias (Table A4 in the Appendix). It is worth mentioning that a total score of nine in three categories is calculated using this numerical bias assessment tool. These three categories include selection, comparability, and exposure/ outcome. Numerical values of four, two, and three are attributed to these categories, respectively.

#### Analysis

A meta-analysis was not feasible due to heterogeneity in adverse reaction types, diverse donor populations, high model variability, and different input data. The results were combined narratively and presented using descriptive statistics. Graphical representations were also created using Datawrapper software [22].

## Results

### Descriptive overview of the included studies and quality assessment

#### Number of included and excluded studies

**Fig. 1:**
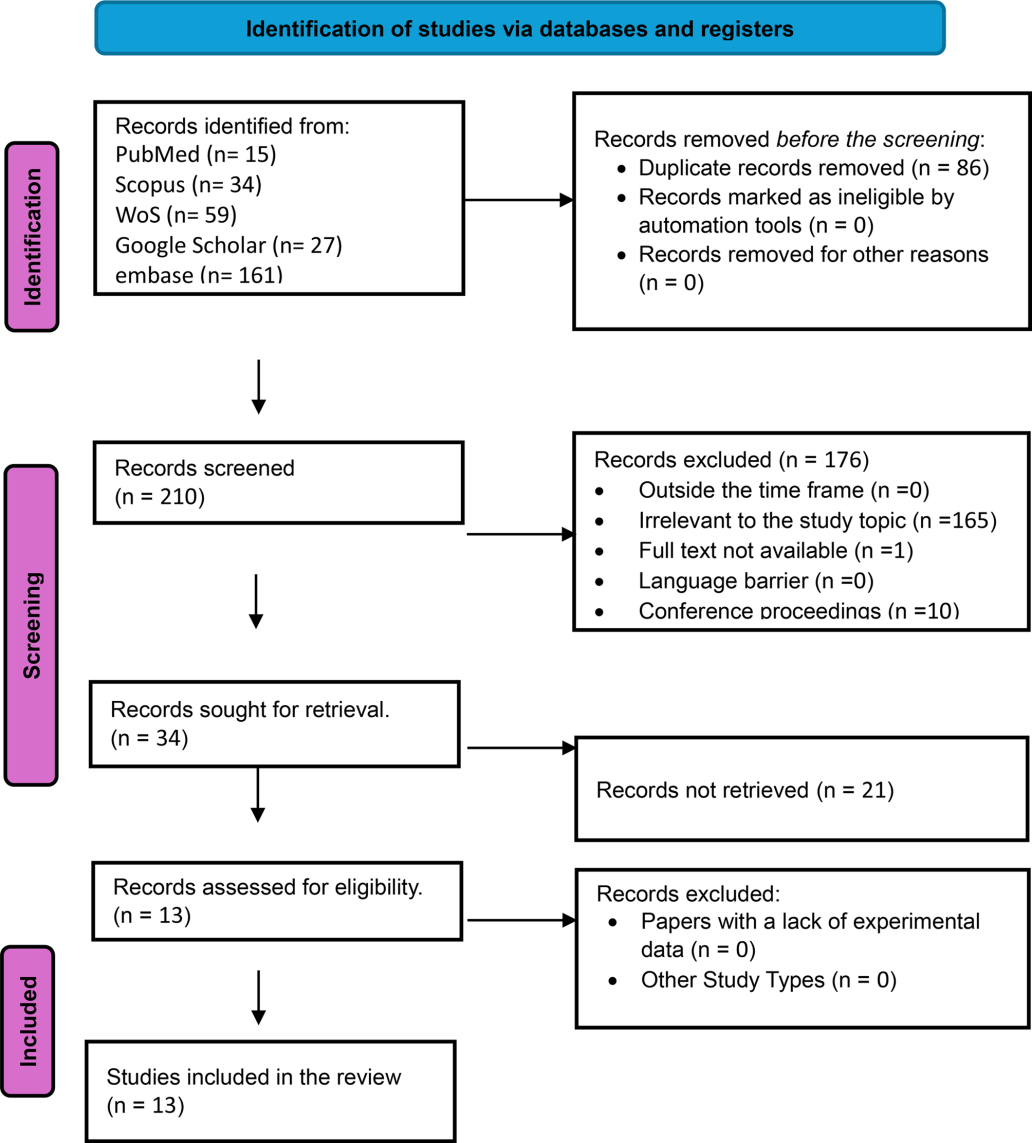
PRISMA 2020 flow diagram of the study retrieval process

The initial database search identified 296 articles. After removing 86 duplicates, 210 articles remained for screening based on title and abstract. Of these, 176 were excluded for failing to meet the predefined inclusion criteria. Full texts of 34 articles were reviewed to assess inclusion criteria more closely, and 13 studies that met all criteria were selected for the final synthesis. Detailed information is presented in the PRISMA flowchart (Fig. 1). All of the included articles were in the fair and good quality range and we did not exclude any studies after the methodological quality assessment (Table A2 in Appendix).

#### Geographical distribution of the studies

**Fig. 2:**
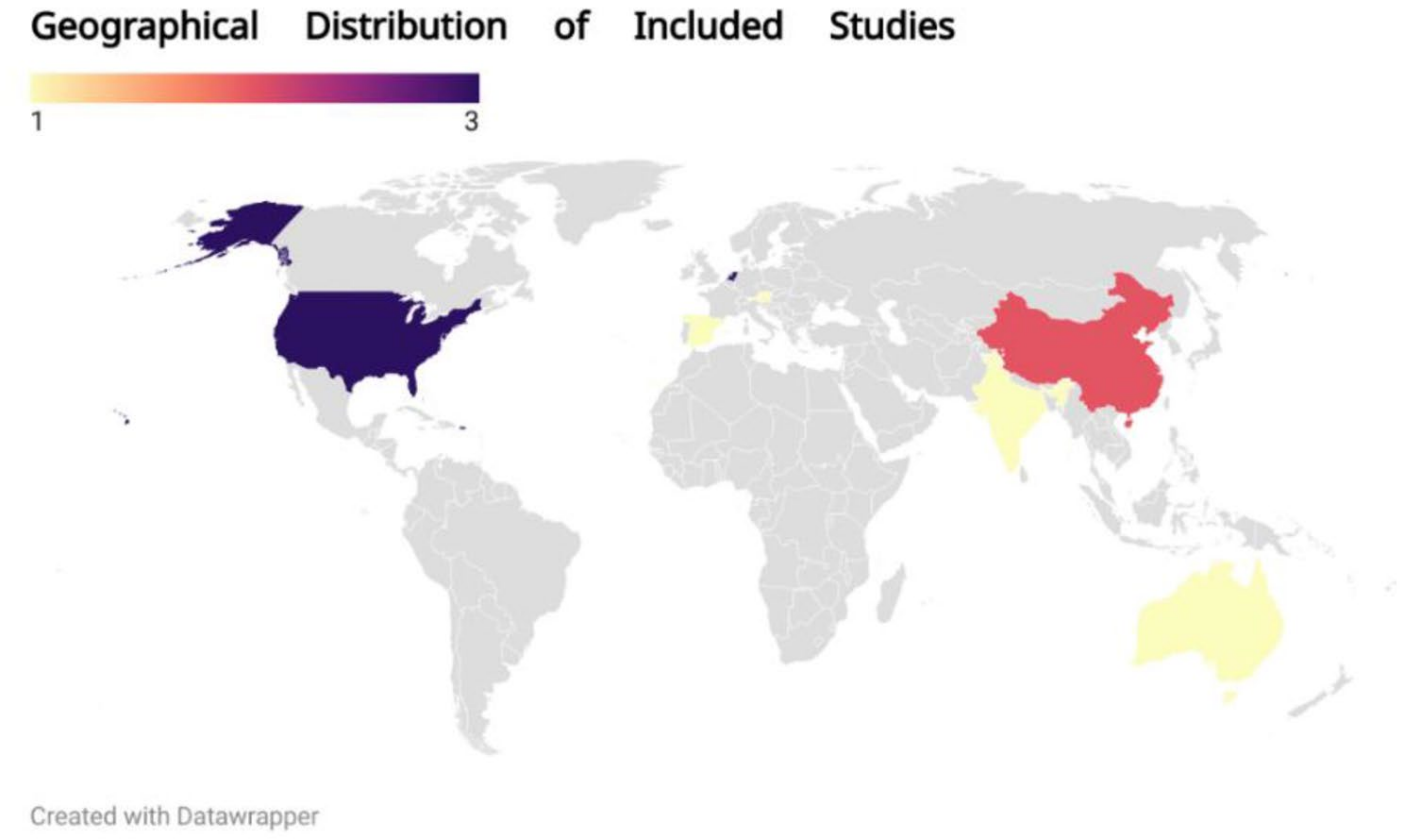
Geographical distribution of included studies

Figure 2 illustrates the global geographical distribution of the included studies. The selected literature predominantly originated from the United States (*n* = 3) and the Netherlands (*n* = 3), followed by China (*n* = 2). Additional single studies were contributed by Hong Kong, Spain, Australia, India, and Austria.

### Overview of models, criteria, data types, population, immediate/delayed classification and blood donation types

Detailed characteristics of the extracted data from the included studies are summarized in Table 1. A detailed explanation of the findings, based on these characteristics, is provided below.

**Fig. 3:**
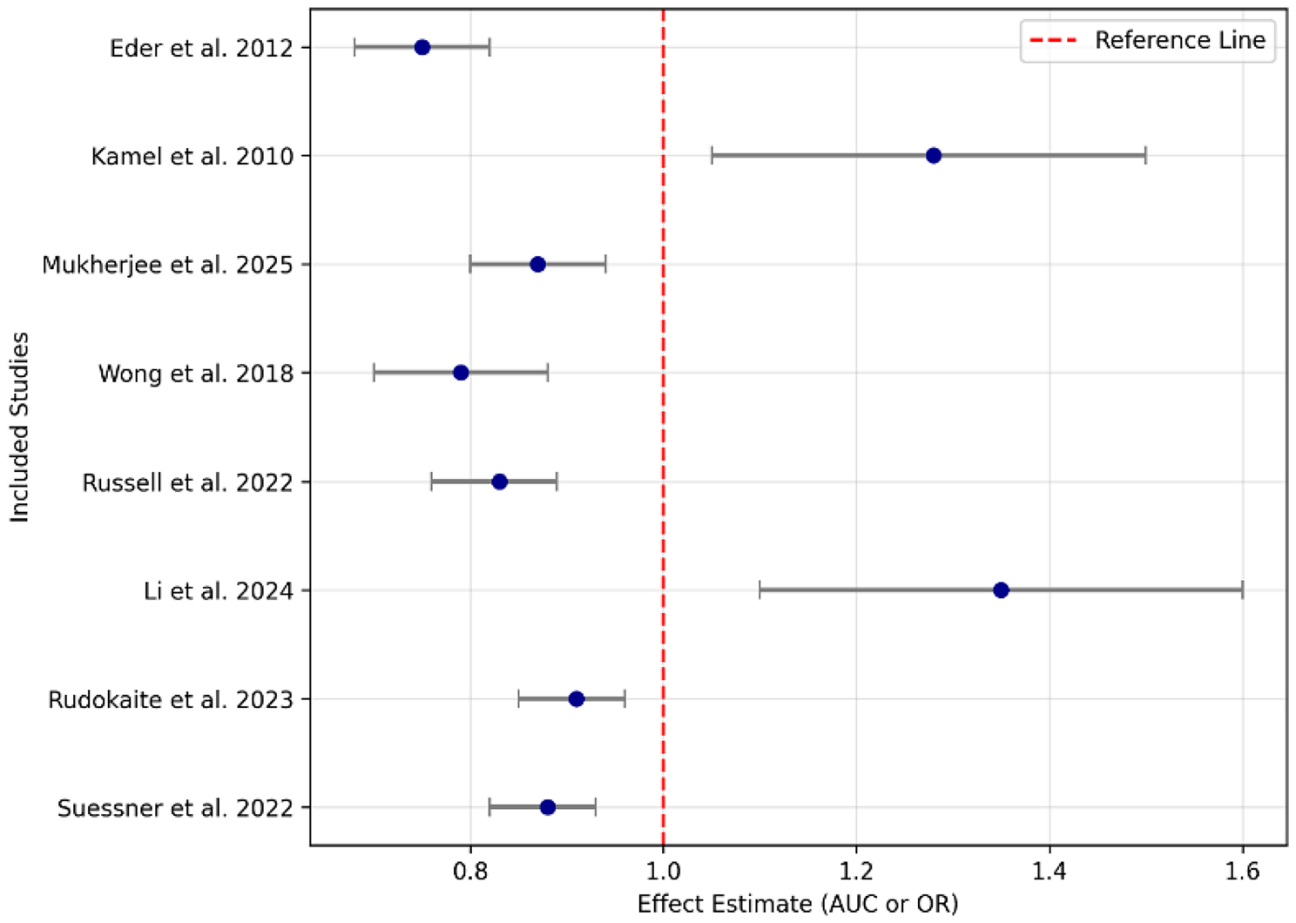
Comparative performance plot of models for predicting ADRs

Due to the infeasibility of conducting a meta-analysis owing to high heterogeneity in model performance metrics and the lack of numerical data on standard errors and confidence intervals, a descriptive approach was employed for the quantitative synthesis. Accordingly, the comparative performance plot (Fig. 3) was constructed descriptively to facilitate visual comparison across studies and AI models.

#### AI models

Analysis of the models used reveals a clear evolution from traditional statistical approaches to more sophisticated machine learning algorithms. While regression models (7 studies) continue to serve as a standard baseline method, a paradigm shift towards machine learning algorithms is evident. Ensemble methods, such as Random Forest (RF) (3 studies) and, mainly, boosting algorithms (used in 4 studies), have been employed to process structured data. Notably, recent studies have emerged that leverage deep learning techniques, such as Artificial Neural Networks (ANNs), Convolutional Neural Networks (CNNs), and Long Short-Term Memory (LSTM) networks, to analyze novel data types, such as videos and thermal images.

#### Evaluation criteria

Among the evaluation criteria, substantial heterogeneity was observed. Discriminatory metrics such as Odds Ratio (OR) and Area Under the Receiver Operating Characteristic Curve (AUC-ROC) were used in five studies and were the most common evaluation metrics. However, a significant gap was noted in the lack of reporting of model calibration metrics in most studies, which are essential for assessing model reliability in clinical applications. Furthermore, a model with a high AUC may not be well-calibrated, resulting in highly confident but incorrect predictions. For instance, overlooking a donor who is genuinely at risk or performing unnecessary interventions on a low-risk donor could lead to wrong decisions. In practice, this could result in inappropriate clinical decisions or wasted resources and costs. In contrast, metrics such as accuracy, recall, and F1 score were predominantly used in newer studies focused on machine learning.

#### Types of input data

In most of the included studies, Textual and Numerical data were used as the input for the models (9 studies). These typically included demographic features, medical histories, and vital signs gathered from blood donor databases. Two studies used video data to analyze donors’ facial expressions or behaviors during blood donation, and one study used thermal imaging of the face as a non-contact method for real-time prediction of VVRs.

#### Immediate/delayed classification

Based on the findings, among a total of 13 studies, nine were classified as immediate reactions, predominantly vasovagal reactions (VVRs; *n* = 7). In contrast, four delayed reactions were reported, encompassing a range of broader health-related outcomes, including iron-related adverse reactions, hospitalization risk due to malignant and benign tumors, mortality, and restless legs syndrome (RLS).

**Table 1:** Findings of included studies

ID	First Author, (Reference)	Year	types of blood donation	Types of ADRs	Immediate / Delayed Reactions	Target population (*N*)	Algorithms or Models Used	Performance Evaluation Metrics	Data Type
1	Kamel, H. [23]	2010	whole blood, apheresis	VVRs	immediate	793,293 donations	regression model	OR	Textual and Numerical
2	Anne, F. [24]	2012	Allogeneic whole blood	syncope	immediate	69,289 donors	statistical model	OR	Textual and Numerical
3	Wong, H. [25]	2018	whole blood	VVRs	immediate	729,347 donations	regression model	OR	Textual and Numerical
4	Russell, W. [26]	2022	whole blood	iron-related adverse reactions	delayed	7279 donations	GBM	PR-AUC	Textual and Numerical
5	Su, S. [27]	2022	whole blood	malignant and benign tumour hospitalization risk	delayed	1,625,599 donors	Regression model	relative risk	Textual and Numerical
6	Suessner, S. [28]	2022	whole blood, pheresis	Fainting	immediate	85,040 donations	RF, ANNs, XGBoost, KNN, Regression model , Support Vector Machine (SVM)	Positive Predictive Value (PPV), Negative Predictive Value (NPV), PR-AUC, F1 score	Textual and Numerical
7	Rudokaite, J. [29]	2023	whole blood	VVRs	immediate	227 donors	Decision tree (DT), RF, XGBoost, ANN	Precision, Recall, F1 score, PR-AUC	video
8	Rudokaite, J. [30]	2023	N/A	VVRs	immediate	193 donors	DT, RF, XGboost, ANNs	Precision, Recall, F1 score, PR-AUC	facial thermal data
9	Li, J. [31]	2024	whole blood	VVRs	immediate	796,764 donations	Statistical model	OR	Textual and Numerical
10	Rahman, M. [32]	2024	whole blood	mortality	delayed	267,357 donors	Statistical model) IPW Marginal Structural Model, TMLE (	Risk ratio	Textual and Numerical
11	Triguero, L. [33]	2025	whole blood	RLS	delayed	129 donors	Regression model	OR	Textual and Numerical
12	Mukherjee, S. [34]	2025	whole blood	VVRs	immediate	2192 donations	Regression model	Incidents of VVRs	Textual and Numerical
13	Rudokaite, J. [35]	2025	whole blood	VVRs	immediate	287 donors	2-Dimensional Convolutional Neural Network (2D-CNNs), LSTM, Gated Recurrent Unit (GRU), Regression model	F1 score, Precision, recall, PR-AUC, Matthews Correlation Coefficient (MCC), Root Mean Square Error (RMSE)	Video

## Discussion

### Key findings

The models employed in the studies predominantly fall into two categories: machine learning and deep learning. The ML approaches primarily consisted of classification and regression algorithms, including, SVM, RF, K-Nearest Neighbors (KNN), and XGBoost. The deep learning models included 2D-CNNs, LSTMs, GRUs, and ANNs. Among the 13 studies reviewed, regression models were the most widely used, appearing in 7 studies. Following that, classical statistical models (3 studies), RF algorithms (3 studies), GBM and XGBoost (4 studies), ANNs (4 studies), DT (2 studies), and SVM and KNN (each in 1 study) were applied.

Among the evaluation metrics, OR and PR-AUC were the most commonly used. Subsequently, F1 Score, Precision, and Recall were the most frequently reported metrics across the studies. Regarding input data types, nine studies used Textual and Numerical data. Two studies used video data to analyze donor behavior or facial expressions during blood donation. Additionally, one study employed facial thermal imaging data to predict adverse reactions.

Adverse reactions were predominantly VVRs, reported in 7 studies. Other adverse reactions, such as syncope, fainting, and RLS, were investigated in individual studies. Additionally, the risk of iron-deficiency complications in donors, the impact of blood donation on hospitalization risk, and the effect of blood donation on mortality were each explored in a single study.

Among the 13 studies, nine were immediate reactions, predominantly vasovagal reactions (VVRs; *n* = 7), while four were delayed reactions, including iron-related adverse events, hospitalization risk due to benign and malignant tumors, mortality, and RLS.

### AI models in ADRs

Based on the results, five studies that used regression models to analyze blood donation data primarily focused on identifying predictors of adverse reactions and on donors’ behavioral or clinical outcomes. Overall, these studies identified age and gender as the most significant influencing variables. Specifically, younger donors and female donors were at a higher risk of experiencing physiological reactions such as VVRs. For example, a study by Shah et al. emphasized the role of risk factors, including first-time donors, female gender, younger age (18–30 years), and lower weight [36]. Similarly, Kamel’s study indicated that lower estimated blood volume prior to donation and limited previous blood donation history increased the likelihood of these reactions [23].

Furthermore, Su’s study reported a positive correlation between blood donation and a reduced risk of benign and malignant tumors, suggesting the potential long-term health benefits of donating blood [27]. A comprehensive review further highlighted the potential cancer-preventing benefits of blood donation, including the elimination of toxins and improvements in overall health [37]. Therefore, these two studies suggest that regular blood donation can help improve the immune system and overall health of the body, benefiting the donor while potentially preventing the onset of dangerous diseases in the future.

Triguero’s findings showed that RLS was more prevalent among female donors [33], while Mukherjee’s study emphasized that the severity of adverse reactions could impede donor return [34]. In general, regression models help determine the key factors, how they interact, and their predictive potential [38]. Thus, the use of regression models has provided deep insights into risk factors and preventive measures to improve donor safety.

In four studies, Gradient-Boosting-Based metrics were used for evaluation and data analysis. The common goal of these studies was to enhance the safety and efficiency of the blood donation process by more accurately predicting potential adverse outcomes based on donors’ biological and behavioral characteristics. XGBoost, a widely recognized implementation of gradient boosting, has demonstrated strong performance in prediction accuracy, interpretability, and classification diversity, making it highly useful for modeling complex systems [39].

In three studies [28–30], neural network models were implemented alongside machine learning algorithms, including RF, DT, and XGBoost. When evaluated using different performance metrics such as accuracy, AUC, sensitivity, and specificity these models yielded varying results. Therefore, no definitive conclusion can be drawn regarding the superiority of one model over others, as differences in data characteristics and outcome definitions can directly influence the reported performance. Moreover, reporting results without accounting for class imbalance, data-splitting strategies (training/validation/test), and hyperparameter-tuning procedures may bias cross-model and cross-study comparisons. Therefore, future research must adopt a unified evaluation framework wherein all models are assessed against a standardized reference dataset, or at the very least, utilizing consistent outcome definitions and fixed validation protocols to ensure reliable and aligned comparisons.

### Input data types in AI models

A series of seminal studies by Rudokaite and colleagues [29, 30, 35] have been instrumental in showcasing the capacity of advanced AI algorithms, particularly Deep Neural Networks, to process complex physiological and behavioral data for predicting VVRs. These studies used video and thermal data, demonstrating the potential of these technologies to identify and accurately predict VVRs.

In the first study [29], the use of video data to analyze physical and emotional reactions revealed that first-time donors and those with a history of previous reactions were at a higher risk. In support of this finding, another study reported that first-time donors had a significantly higher risk of adverse reactions, with an increased OR of 6.40 [40]. The second study [30] used facial thermal data obtained via infrared imaging to detect subtle temperature changes on the face before the donation process, serving as latent indicators of physiological and emotional reactions. The ANN and XGBoost models achieved high performance in predicting these reactions (F1 = 0.88). While various studies have used facial thermal data [41–43], represents a novel application of this technology.

The third study [35] focused on the use of 2D-CNN and LSTM deep neural networks to analyze videos of blood donors, showing that the combined ResNet152-LSTM model achieved the best performance in classifying reactions. In contrast, the GRU model was better suited for real-time applications due to its higher speed. Furthermore, shorter videos (5 s) provided similar or even better accuracy than longer videos. Collectively, these studies underscore that multimodal data streams, processed via sophisticated deep learning architectures, present a promising new paradigm for both pre-donation risk stratification and real-time monitoring.

### Evaluation metrics used in models

Among the studies reviewed, OR and PR-AUC were the most commonly used evaluation metrics, each appearing in 5 studies. Following these, F1 score (4 studies), Precision, and Recall (each used in 3 studies) were also frequently applied. Other metrics, such as Relative Risk, Risk Ratio, PPV, NPV, MCC, and RMSE, were reported in only one study. The frequency of VVRs as an evaluation metric was also reported in one study. The OR is a fundamental statistical measure widely used in public health and medical research to assess associations between exposures and outcomes by comparing odds between exposed and unexposed groups [44]. In the reviewed studies, OR was used to analyze and predict the risk of physiological reactions in blood donors, thereby identifying patterns that influence these reactions by examining various factors. The ROC Curve (Receiver Operating Characteristic Curve) plots the true positive rate (TPR) against the false positive rate (FPR), providing an overall diagnostic performance evaluation and aiding in selecting the optimal threshold [45]. This metric was used in studies employing both machine learning and deep learning models to assess performance.

### Specific ADRs observed in studies

The synthesized literature indicates that immediate-onset reactions, particularly VVRs and syncope, constitute the majority of ADRs in blood donors. This aligns with findings from a large-scale study where VVRs accounted for 87.1% of all reported adverse events [46]. Some studies have explored specific ADRs such as syncope and fainting in blood donors. The study [24] indicates that syncope after the first blood donation significantly reduces donor return rates (18%) compared to those who did not react (35%). In support of this, a systematic review [47] on identifying risk factors for syncope in healthy blood donors demonstrated that adverse reactions can reduce the likelihood of future blood donations. In another study, Dipaola and colleagues developed natural language processing algorithms to automatically identify patients with syncope from emergency department medical records, achieving a sensitivity of 92.2% [48]. Integrating AI models with blood donors’ electronic health records (EHRs) could help predict and prevent syncope before Donation. The study [28], which used individual characteristics and machine learning algorithms, showed that syncopal events in blood donors could be predicted with high accuracy. In addition to using machine learning models to predict syncopal events, other novel technologies have been employed. Wong et al. proposed a thermal-imaging-based surveillance system for detecting fainting events, achieving 96.15% accuracy in low-light conditions and 86.19% in indoor environments.

While less frequent, delayed reactions warrant careful consideration due to their potential for more serious long-term health implications. For example, The study [33] demonstrated that the prevalence of RLS among blood donors is 14.1%, which is higher than that in the general population and other cohorts. this study found that the prevalence of RLS is higher among female blood donors than among males. The sample population in this study consisted of 129 donors, limiting the generalizability of the results to a larger population. Further studies with larger sample sizes are needed for more robust validation.

Although another study [32] found no significant association between frequent blood donation (regular donors) and mortality, this finding should be interpreted with caution, as differences in donors’ baseline characteristics, potential confounding factors, and study-design limitations may have influenced the results. The potential for confounding variables and inherent limitations in study design underscore the need for rigorous, large-scale prospective studies to definitively assess the long-term safety profile of regular blood donation.

### Strengths

The strengths of this study lie in several key aspects. Firstly, a diverse range of databases was utilized for resource searching, which significantly contributed to a more comprehensive identification of relevant studies. In this research, various dimensions of ADRs were thoroughly examined, including the type of reaction, the models used, and their evaluation criteria, the types of data employed in the studies, and the types of blood donation. These elements were systematically analyzed and discussed. This study offers a novel synthesis for hematology professionals regarding the scope and application of AI and machine learning models in identifying and preventing adverse blood donation events. By reviewing this study, hematologists will be able to understand the current status of AI model implementation and, by recognizing the existing strengths and weaknesses, propose practical solutions to improve the quality of the blood donation process and increase donor participation in future research. Additionally, medical informatics specialists can enhance donor safety through intelligent systems by analyzing models, identifying their strengths and weaknesses, and applying them in blood donation environments. The findings of this study provides an evidence-based foundation for health policymakers and blood transfusion organization managers in developing new strategies to enhance donor safety and improve their overall experience.

### Challenges and research gaps

Despite the study’s valuable capabilities, it faces several challenges. One of the most significant challenges is the lack of integration of any of the AI models presented in the studies with the EHR of blood donors. Most studies were conducted independently and limited to data collected within specific time frames, focusing solely on research rather than real-world applications.

Moreover, most studies have focused on metrics such as accuracy and sensitivity using ROC-AUC. In contrast, calibration metrics, including slope, intercept, Expected Calibration Error (ECE), Brier score, and Decision Curve Analysis (DCA), have been underexplored. This focus can impact the clinical applicability of the models. Additionally, clinical and economic effects have not been sufficiently addressed in the studies. These evaluations play a crucial role in assessing the actual impact of AI models on improving therapeutic outcomes and reducing costs. They can provide valuable insights for clinical decision-making and health policy development. Given that many models are evaluated primarily on technical criteria, neglecting clinical and economic factors may hinder proper assessment of their real-world applicability in healthcare settings.

The research gap lies in the underutilization of deep learning models for identifying and managing sudden changes in blood donors’ condition. These models can analyze physiological data (such as heart rate and blood pressure) and behavioral data (such as facial and head movements) to simulate critical changes and send alerts through Clinical Decision Support Systems (CDSS) to the medical team.

Furthermore, some studies have focused on adverse reactions in first-time blood donors [34], while others have examined reactions in repeat donors [26]. This inconsistency across studies may introduce bias when generalizing the results to all blood donors. As a result, extrapolating findings to the entire donor population may not be accurate and could lead to misleading conclusions.

Another challenge lies in the legal ambiguities surrounding liability for errors made by AI models when deployed in a blood donation environment. For instance, if an adverse reaction occurs in a donor and the AI model fails to predict it, it remains unclear who is accountable. Conversely, if the model predicts a reaction that does not occur, liability still arises [49]. Responsible AI requires identifying the relative responsibility of all stakeholders involved in the design, development, deployment, and use of AI systems [50]. Determining responsibility necessitates precise methods that can identify what or who is accountable for the behavior and outcomes of the AI system, which is closely related to the identification of causal elements [51]. In healthcare contexts, explainable AI techniques can help meet the transparency, fairness, accountability, and safety needs for responsible AI by making the model’s internal processes transparent [52].

Research shows that 8.3–8.5% of blood donors experience adverse reactions, with most (67%) occurring after leaving the donation center [53]. Another research gap identified in this study is the lack of follow-up for donors who experience reactions at least 15 min after Donation, off-site. Therefore, it is recommended that donor follow-up be conducted via phone calls [54], remote monitoring, and the recording of adverse reactions in personal electronic health records [55] or portals [56], ensuring accurate collection, completion, and management of adverse reactions.

Another gap in the study is that none of the included studies were designed in a multimodal approach. This points to a lack of integration of data and methods in the existing research, which could offer new opportunities for more comprehensive and accurate analysis, particularly in complex areas such as behavioral and physiological data analysis. As the diversity of data from various sources, such as text, Numerical, images, videos, and audio, expands, there is an increasing need for advanced multimodal data analysis techniques [57].

### Limitations and future recommendations

Despite searching five reputable databases, only a limited number of studies met the eligibility criteria. Relevant studies may exist in other databases or in languages other than English that were not captured. Therefore, it is recommended that future research expand the search to include a broader range of databases and consider studies published in multiple languages.

A review of the studies revealed that none of the models were consistently implemented in a practical, operational manner before or during clinical examinations or blood donation. Hence, it is recommended that future research integrate AI models with donors’ electronic health records to enable real-time identification and management of potential reactions by accessing the donors’ clinical history.

It is also recommended that future studies utilize ontologies for AI models in the context of all types of ADRs, to enable systematic documentation and optimal use of the data [58, 59].

## Conclusion

Overall, the available evidence indicates that machine learning and AI-based approaches demonstrate considerable potential for identifying and predicting ADRs. However, the current body of literature is not yet sufficient to establish a robust evidence base for the widespread integration of these models into clinical or operational decision-making. The reviewed studies exhibited substantial heterogeneity regarding study design, outcome definitions, and evaluation metrics. Furthermore, their generalizability and clinical interpretation are frequently constrained by limited real-world implementation, inherent risks of bias, unclear reference standards, and a conspicuous lack of external validation. Accordingly, it is imperative that future research prioritizes rigorous methodological designs, standardized reporting guidelines, harmonized evaluation metrics, and extensive external validation to definitively ascertain the efficacy and reliability of these predictive models. This study has identified several research gaps. The first gap is the lack of deep learning models for identifying and managing sudden changes in donor status in real time. These models could send alerts by analyzing physiological and behavioral data, allowing timely interventions. Another gap is the lack of accurate follow-up for donors experiencing delayed reactions, especially when reactions occur off-site and are not reported by the donor. Additionally, studies require a multimodal approach (processing various data types such as images, text, Numerical and audio), which has not been used in any of the studies. Furthermore, the evaluation of AI models has mainly focused on technical metrics, with their clinical and economic impacts in real-world settings not assessed. Legal, ethical, and data privacy considerations must also be addressed before implementing these models in blood donation processes.

## Electronic supplementary material

Below is the link to the electronic supplementary material.

Supplementary Material 1

## Data Availability

Data available on request from the authors.

## Abbreviations

ADRs: Adverse Donor Reactions
ATRs: Adverse Transfusion Reactions
VVRs: Vasovagal Reactions
PR-AUC: Precision-Recall Area Under the Curve
AI: Artificial Intelligence
PICO/PECO: Population, Intervention/Exposure, Comparator, Outcome
PRISMA: Preferred Reporting Items for Systematic Reviews and Meta-Analyses
PROSPERO: International Prospective Register of Systematic Reviews
NOS: The Newcastle-Ottawa Scale
MeSH: Medical Subject Headings
RF: Random Forest
ANNs: Artificial Neural Networks
CNNs: Convolutional Neural Networks
LSTM: Long Short-Term Memory
GBM: Gradient Boosting Machine
XGBoost: Extreme Gradient Boosting
KNN: K-Nearest Neighbors
SVM: Support Vector Machine
DT: Decision Tree
2D-CNN: 2-Dimensional Convolutional Neural Network
GRU: Gated Recurrent Unit
MCC: Matthews Correlation Coefficient
RMSE: Root Mean Square Error
IPW: Inverse Probability Weighting
TMLE: Targeted Maximum Likelihood Estimation
PPV: Positive Predictive Value
NPV: Negative Predictive Value
OR: Odds Ratio
RR: Relative Risk
EHR: Electronic Health Record
RLS: Restless Leg Syndrome
DCA: Decision Curve Analysis
EPR: Electronic Patient Record
CDSS: Clinical Decision Support System
ECE: Expected Calibration Error
TPR: True positive rate
FPR: False positive rate
