# Supplementary material for: Artificial Intelligence-based predictive models for adverse blood donor reactions: a systematic review of immediate and delayed events and clinical data approaches
Source: BMC Med Inform Decis Mak. 2026 May 25;26:271. doi: 10.1186/s12911-026-03584-0 (PMC13383129; doi:10.1186/s12911-026-03584-0)
Supplement: Supplementary file 1 — Supplementary Material 1 [file 12911_2026_3584_MOESM1_ESM.docx]

**Table A1**: search strategy

| **DB** | **Search strategy** |
| --- | --- |
| PubMed | ("Artificial Intelligence"[MeSH Terms] OR "Artificial Intelligence"[Title/Abstract] OR "AI"[Title/Abstract] OR "Machine Learning"[MeSH Terms] OR "machine learning*"[Title/Abstract] OR "Natural Language Processing"[MeSH Terms] OR "neural networks, computer"[MeSH Terms] OR "neural network*"[Title/Abstract] OR "Expert Systems"[MeSH Terms] OR "expert system*"[Title/Abstract] OR "decision support systems, clinical"[MeSH Terms] OR "clinical decision support system*"[Title/Abstract] OR "CDSS"[Title/Abstract] OR "Data Mining"[Title/Abstract] OR "intelligent system*"[Title/Abstract]) AND ("Blood Donors"[MeSH Terms] OR "Blood Donor"[Title/Abstract] OR "donor blood"[Title/Abstract] OR "donors blood"[Title/Abstract] OR "Blood Donation"[MeSH Terms] OR "Blood Donations"[Title/Abstract] OR "donation blood"[Title/Abstract] OR "donating blood"[Title/Abstract] OR "Plasma Donation"[Title/Abstract] OR "donation plasma"[Title/Abstract] OR "donations plasma"[Title/Abstract] OR "Plasma Donations"[Title/Abstract] OR "donation platelet"[Title/Abstract] OR "Platelet Donations"[Title/Abstract]) AND ("Donor reaction"[Title/Abstract] OR "adverse effect*"[Title/Abstract] OR "side effect*"[Title/Abstract] OR "Donor adverse event"[Title/Abstract] OR "risk*"[Title/Abstract] OR "hazard*"[Title/Abstract] OR "complication*"[Title/Abstract] OR "adverse event*"[Title/Abstract] OR "Donation reaction"[Title/Abstract] OR "Donor complication"[Title/Abstract] OR "Donor response"[Title/Abstract]) |
|  | ( TITLE-ABS ("Artificial Intelligence" OR "AI" OR "Machine Learning" OR "machine learning" OR "Natural Language Processing" OR "neural networks, computer" OR "neural network" OR "Expert Systems" OR "expert system" OR "decision support systems, clinical" OR "clinical decision support system" OR "CDSS" OR "Data Mining" OR "intelligent system" ) AND ( TITLE-ABS ( "Blood Donors" OR "Blood Donor" OR "donor blood" OR "donors blood" OR "Blood Donation" OR "Blood Donations" OR "donation blood" OR "donating blood" OR "Plasma Donation" OR "donation plasma" OR "donations plasma" OR "Plasma Donations" OR "donation platelet" OR "Platelet Donations" ) AND ( TITLE-ABS ( "Donor reaction" OR "adverse effect" OR "side effect" OR "Donor adverse event" OR "risk" OR "hazard" OR "complication" OR "adverse event" OR "Donation reaction" OR "Donor complication" OR "Donor response" ) ) ) ) |
| Web of Science | (TS= ( "Artificial Intelligence" OR "AI" OR "Machine Learning" OR "machine learning" OR "Natural Language Processing" OR "neural networks, computer" OR "neural network" OR "Expert Systems" OR "expert system" OR "decision support systems, clinical" OR "clinical decision support system" OR "CDSS" OR "Data Mining" OR "intelligent system" ) AND (TS= ( "Blood Donors" OR "Blood Donor" OR "donor blood" OR "donors blood" OR "Blood Donation" OR "Blood Donations" OR "donation blood" OR "donating blood" OR "Plasma Donation" OR "donation plasma" OR "donations plasma" OR "Plasma Donations" OR "donation platelet" OR "Platelet Donations" ) AND (TS= ( "Donor reaction" OR "adverse effect" OR "side effect" OR "Donor adverse event" OR "risk" OR "hazard" OR "complication" OR "adverse event" OR "Donation reaction" OR "Donor complication" OR "Donor response" ) ) ) ) |
| Embase | ('artificial intelligence'/exp OR 'artificial intelligence' OR 'ai' OR 'machine learning'/exp OR 'machine learning' OR 'natural language processing'/exp OR 'natural language processing' OR 'neural networks, computer'/exp OR 'neural networks, computer' OR 'neural network'/exp OR 'neural network' OR 'expert systems'/exp OR 'expert systems' OR 'expert system'/exp OR 'expert system' OR 'decision support systems, clinical'/exp OR 'decision support systems, clinical' OR 'clinical decision support system'/exp OR 'clinical decision support system' OR 'cdss' OR 'data mining'/exp OR 'data mining' OR 'intelligent system') AND ('blood donors'/exp OR 'blood donors' OR 'blood donor'/exp OR 'blood donor' OR 'donor blood' OR 'donors blood' OR 'blood donation'/exp OR 'blood donation' OR 'blood donations' OR 'donation blood' OR 'donating blood' OR 'plasma donation' OR 'donation plasma' OR 'donations plasma' OR 'plasma donations' OR 'donation platelet' OR 'platelet donations') AND ('donor reaction' OR 'adverse effect'/exp OR 'adverse effect' OR 'side effect'/exp OR 'side effect' OR 'donor adverse event' OR 'risk'/exp OR 'risk' OR 'hazard'/exp OR 'hazard' OR 'complication'/exp OR 'complication' OR 'adverse event'/exp OR 'adverse event' OR 'donation reaction' OR 'donor complication' OR 'donor response') |
| Google Scholar | donor AND adverse donor reaction AND artificial intelligence |

**Table A2:** Characteristics of included studies

| **ID** | **First Author, (Reference)** | **Year** | **Study Type** | **Country** | **The aim of the study** |
| --- | --- | --- | --- | --- | --- |
| 1 | Kamel, H. (1) | 2010 | retrospective study | USA | The aim of this study is to identify the risk factors associated with delayed donor reactions (DRs). |
| 2 | Anne, F. (2) | 2012 | Case-control studies | USA | In this study, we assessed the risk of recurrent syncope among returning blood donors. |
| 3 | Wong, H. (3) | 2018 | retrospective study | Hong Kong | Identifying individuals at risk of vasovagal reactions |
| 4 | Russell, W. (4) | 2022 | Retrospective study | USA | The aim of the study is to develop and evaluate multiclass prediction models to estimate the risk of iron-related adverse outcomes in blood donors at their subsequent donation. Another aim of the study is to compare the performance of the models with and without the use of two non-routine biomarkers (ferritin and soluble transferrin receptor) and to analyze individual risk trajectories for each donor based on the time interval between donations. |
| 5 | Su, S. (5) | 2022 | Cohort study | China | This study aims to identify the relationship between blood donation and malignant and benign tumour hospitalization risk. |
| 6 | Suessner, S. (6) | 2022 | retrospective  study | Austria | The aim of this study is to identify factors associated with syncope in blood donors in order to prevent adverse events during the blood donation process. |
| 7 | Rudokaite, J. (7) | 2023 | observational study | Netherlands | This study aims to investigate whether a blood donors’ unconscious facial microexpressions in the waiting room, prior to actual blood donation, can be used to predict who will experience a VVR later, during the donation. |
| 8 | Rudokaite, J. (8) | 2023 | experimental study | Netherlands | This study aims to investigate whether facial temperature profiles measured in the waiting room, prior to a blood donation, can be used to classify who will and will not experience VVR during the donation |
| 9 | Li, J. (9) | 2024 | descriptive cross-sectional study | China | Study aim: To provide and analyze regional hemovigilance data in Chongqing, China, to report the prevalence of vasovagal reactions (VVR) and identify associated risk factors. |
| 10 | Rahman, M. (10) | 2024 | longitudinal cohort study | Australia | The aim of this study was to examine the relationship between regular high-frequency blood donation and mortality risk in Australian blood donors aged 45 years and older, using methods to minimize the healthy donor effect (HDE). |
| 11 | Triguero, L. (11) | 2025 | prospective cohort study | Spain | This study aims to assess the prevalence of RLS in Spanish blood donors and determined its potential correlation with iron metabolism parameters. |
| 12 | Mukherjee, S. (12) | 2025 | cohort study | India | This study aims to find out the effectiveness of applied muscle tension in reducing vasovagal events in first-time blood donors. |
| 13 | Rudokaite, J. (13) | 2025 | experimental study | Netherlands | The aim of this study was to investigate the possibility of predicting vasovagal reaction (VVR) levels through the analysis of facial video sequences recorded during the blood donation process. |

**Table A3.** Description of the findings reported in the eligible studies

| **ID** | **Type of blood Donation** | **Types of Adverse Donor Reactions** | **Immediate / Delayed Reactions** | **Target population (N)** | **Algorithms or Models Used** | **Performance Evaluation Metrics** | **Data Type** | **Main findings** |
| --- | --- | --- | --- | --- | --- | --- | --- | --- |
| 1 | whole blood, apheresis | vasovagal reactions | immediate | 793,293 donations | regression model | Odds ratio | Textual and Numerical | Multivariable logistic regression identified that EBV, age, and donation history were highly associated with IRs, corroborating our previous study,10 and identified sex, race/ethnicity, donation site, and donation history as significant contributors to DRs. Predonation donor blood volume is inversely associated with the likelihood of either an IR or DR. The observation that sex is a better predictor of a DR than an IR is novel. Female sex has been consistently, but with varying magnitude, considered a risk factor for vasovagal reactions after blood donation.1,10,19-24 In this study, female sex was shown only to be a significant predictor for DRs. The second highest OR in the multivariable analysis comparing DRs to no reactions was seen for women. This is striking because of the lack of sex impact on IRs. The issue is made more important when we look at the multivariable model comparing DRs to IRs. The odds of a reacting female donor having a DR were more than three times higher than the odds of a reacting male donor. |
| 2 | Allogeneic whole blood | syncope | immediate | 69,289 donors | statistical model | Odds ratio | Textual and Numerical | Syncope after a first whole blood donation significantly reduced the frequency of return donation (18%), compared to either presyncopal symptoms (27%; p < 0.0001) or no reaction (35%; p < 0.0001). Among novice donors who returned to donate, syncope was more likely among donors who had any reaction (0.8%) or syncope (3.5%) at their first donation, compared to donors who had no reaction (0.3%; p < 0.0001). Syncope at a first donation identified only 2% (19 of 1062) of syncopal reactions among returning donors. For active, repeat donors who experienced syncope in 2009, a history of prior reactions had no effect on the likelihood of return donation or recurrent syncope. |
| 3 | whole blood | vasovagal reactions | immediate | 729,347 donations | regression model | Odds ratio | Textual and Numerical | he observed incidence rate of vasovagal reaction during and after whole blood donation was around 1.5%. Our analysis indicated that young age was a significant independent variable in predicting vasovagal reactions which is consistent with many other studies. first-time donors aged less than 20 had a reaction rate of 4.3%, while those aged at or above 50 got a rate of 0.3% only. |
| 4 | whole blood | iron- related adverse reactions | delayed | 7279 donations | GBM | PR-AUC | Textual and Numerical | This analysis of 7279 index donations from the RISE study found that risk of iron-related adverse outcomes at follow-up visits can be estimated as a function of the inter-donation interval. Including ferritin as a predictor improved risk estimation, particularly for estimating risk of low and absent iron donations, but AUC was above 70% for all outcomes even with standard biomarkers. For most donors, estimated risk decreased precipitously for longer donation intervals, suggesting that longer minimum donation intervals would prevent some cases of donation-associated iron deficiency and hemoglobin deferrals. For other donors, estimated risk of an adverse outcome remained over 90% even for a 250-day donation interval. |
| 5 | whole blood | malignant and benign tumour hospitalization risk | delayed | 1,625,599 donors | Regression model | relative risk | Textual and Numerical | Malignant Tumours: Hospitalization prevalence of malignant tumours was significantly lower in male blood donors compared to non-donors (0.21% vs. 0.28%; P < 0.05, RR = 0.82, 95% CI: 0.75–0.92) and also lower in female donors (0.31% vs. 0.40%; P < 0.01). Benign Tumours: In males, the most common benign neoplasm occurred in the major salivary glands, with no significant difference in risk between donors and non-donors. However, the risk of hemangioma, lymphangioma, and other skin neoplasms was significantly lower in donors (RR = 0.75 [0.51–0.89] and 0.79 [0.62–0.94], respectively). |
| 6 | whole blood, pheresis | Fainting | immediate | 85,040 donations | Random Forest, ANN, XGBoost,  KNN, Regression model  , SVM | PPV, NPV, PR-AUC, F1 score | Textual and Numerical | The main result of our study is that fainting reactions during blood donations can be predicted with similar good precision by seven mathematically different machine learning algorithms using the properties of the blood donor and local weather reports. |
| 7 | whole blood | vasovagal reactions | immediate | 227 donors | Decision tree,  Random Forest, XGBoost, ANN | Precision, Recall, F1 score, PR-AUC | video | The results showed that first-time donors and those with a previous experience of VVR exhibited higher levels of physical and emotional reactions during donation compared to the control group. Furthermore, it was found that pre-donation self-reported scores alone were insufficient to predict the occurrence of VVR, as models based solely on these data were limited in accurately identifying donors with high VVR levels (F1 = 0.77). |
| 8 | N/A | vasovagal reactions | immediate | 193 donors | Decision tree, Random Forest, XGboost, ANN | Precision, Recall, F1 score, PR-AUC | facial thermal data | The study demonstrated that adverse low and high emotional and physical reactions during blood donation can be predicted based on covert, automatic physiological processes occurring in anticipation of the procedure. Neural network and XGBoost models trained on anticipatory facial temperature fluctuations, measured using infrared thermal imaging in the waiting room, performed well, achieving an F1 score of 0.88 for classifying the low VVR group. |
| 9 | whole blood | Vasovagal reactions | immediate | 796,764 donations | Statistical model | Odds ratio | Textual and Numerical | RIR of complications was 8.25‰ from January 2020 to December 2022. This number was considerably lower than the 20.8‰–24.3 ‰ reported in the AABB Donor Hemovigilance Report for 2012–2017. the incidence rate of ADR associated with WB was 8.76‰, which is higher than the national data of 3.8‰ from July 2019 to December 2021 in China; however, the rate of ADR related to PA was 1.88‰, which was lower than the national level of 2.2‰ during the corresponding period. Regarding the 3-year incidence rate of VVR, we found that WB donation was associated with 8.69‰, and PA was responsible for 1.02‰. Different from another study which reported that the incidence rates for VVR in WB donations range from 10‰–125‰ and from 1.6‰ to 41.7‰ in PA collections |
| 10 | whole blood | mortality | delayed | 267,357 donors | Statistical model) IPW Marginal Structural Model, TMLE ( | Risk ratio | Textual and Numerical | We selected 4750 (64.7%) low-frequency and 2588 (35.3%) high frequency donors in the analyses. A total of 69 (1.5%) from the low-frequency and 45 (1.7%) donors from the regular high-frequency group died during the 7-year follow-up period. We did not find any statistically significant association between regular high-frequency blood donation and mortality (IPW RR = 0.98 95% CI 0.68, 1.28). TMLE model also showed similar results to IPW (RR = 0.97 95% CI 0.80, 1.16). Time-varying TMLE did not find any significant association between high-frequency donation and all-cause mortality either (RR = 0.98 95% 0.74, 1.29). |
| 11 | whole blood | restless legs  syndrome | delayed | 129 donors | Regression model | Odds ratio | Textual and Numerical | Data from the ‘‘RLS Epidemiology, Symptoms, and Treatment (REST)’’ showed that RLS symptoms were present in approximately 7% of the general population. The estimated prevalence of RLS in the Spanish REST sub-sample was 5.5%. 3 This percentage increased up to 11.6% in patients recruited in a primary health care center with different complaints 5 and is similar to the 14.1% observed in the participants recruited in our study and in other European countries. 4,23 This prevalence is higher in women blood donors, in our study compared to men (ratio 3:1 at inclusion and nearly 2:1 at follow up). |
| 12 | whole blood | vasovagal reactions | immediate | 2192 donations | Regression model | Incidences of vasovagal reactions | Textual and Numerical | Incidences of VVRs as adverse donor reactions in whole blood donations significantly affect the donors’ return behavior. The greater the number and severity of adverse reactions, the less likely donors are to return for a donation. As a result, efforts are made to reduce the incidence of adverse events (AEs) related to blood donation. The standard method to prevent VVRs is ingesting plenty of fluid before blood donation. |
| 13 | whole blood | vasovagal reactions | immediate | 287 donors | 2D-CNN, LSTM, GRU, Regression model | F1 score, Precision, recall, PR-AUC, MCC, RMSE | video | The results indicated that the best performance in the classification task was achieved using the ResNet152-LSTM model, although the difference compared to the GRU and Xception models was minimal. In the regression task, the GRU model demonstrated superior performance and, due to its higher processing speed, is considered more suitable for real-time applications. It was also found that shorter video sequences (5 seconds) yielded comparable or even slightly better accuracy. Specifically, the highest F1 score (0.74) for the high VVR group was achieved using a 5-second video input, while the lowest RMSE (2.56) was obtained with the Xception model using a 10-second input. |

**Table A4**: Newcastle-Ottawa Scale (NOS) bias risk assessment of the study.

| ID | The first author (reference) | Selection  (out of 4) | Comparability  (out of 2) | Exposure/Outcome  (out of 3) | Total  (Out of 9) |
| --- | --- | --- | --- | --- | --- |
| 1 | Kamel, H. (1) | 3 | 2 | 3 | Good quality |
| 2 | Anne, F. (2) | 4 | 2 | 3 | Good quality |
| 3 | Wong, H. (3) | 3 | 2 | 3 | Good quality |
| 4 | Russell, W. (4) | 2 | 2 | 2 | Fair quality |
| 5 | Su, S. (5) | 4 | 1 | 3 | Good quality |
| 6 | Suessner, S. (6) | 3 | 2 | 3 | Good quality |
| 7 | Rudokaite, J. (7) | 3 | 2 | 2 | Good quality |
| 8 | Rudokaite, J. (8) | 4 | 1 | 3 | Good quality |
| 9 | Li, J. (9) | 3 | 2 | 3 | Good quality |
| 10 | Rahman, M. (10) | 2 | 2 | 2 | Good quality |
| 11 | Triguero, L. (11) | 3 | 2 | 3 | Good quality |
| 12 | Mukherjee, S. (12) | 3 | 2 | 3 | Good quality |
| 13 | Rudokaite, J. (13) | 2 | 2 | 2 | Fair quality |

Note: Good quality: 3 or 4 stars in selection domain AND 1 or 2 stars in comparability domain AND 2 or 3 stars in exposure/outcome domain; Fair quality: 2 stars in selection domain AND 1 or 2 stars in comparability domain AND 2 or 3 stars in exposure/outcome domain; Poor quality: 0 or 1 star in selection domain OR 0 stars in comparability domain OR 0 or 1 stars in exposure/outcome domain.

**References:**

1. Kamel H, Tomasulo P, Bravo M, Wiltbank T, Cusick R, James RC, et al. Blood donors and blood collection: delayed adverse reactions to blood donation. Transfusion. 2010;50(3):556-65.

2. Eder AF, Notari IV EP, Dodd RY. Do reactions after whole blood donation predict syncope on return donation? Transfusion. 2012;52(12):2570-6.

3. Wong Hk, Chu CCy, Lau Cw, Leung JNs, Lee IYm, Lee Ck. Vasovagal reaction in blood donors: prediction and its impact on donor return. ISBT Science Series. 2018;13(4):421-8.

4. Russell WA, Scheinker D, Custer B. Individualized risk trajectories for iron-related adverse outcomes in repeat blood donors. Transfusion. 2022;62(1):116-24.

5. Su S, Ma T, Sun Y, Guo L, Su X, Wang W, et al. Association between Blood Donation and Malignant and Benign Tumour Risk: A Population-Based Study of 3.4 Million Participants in China. J Oncol. 2022;2022:7647431.

6. Suessner S, Niklas N, Bodenhofer U, Meier J. Machine learning-based prediction of fainting during blood donations using donor properties and weather data as features. BMC Med Inform Decis Mak. 2022;22(1):222.

7. Rudokaite J, Ertugrul IO, Ong S, Janssen MP, Huis In 't Veld E. Predicting Vasovagal Reactions to Needles from Facial Action Units. J Clin Med. 2023;12(4).

8. Rudokaite J, Ong LLS, Onal Ertugrul I, Janssen MP, Huis In 't Veld EMJ. Predicting vasovagal reactions to needles with anticipatory facial temperature profiles. Sci Rep. 2023;13(1):9667.

9. Li J, Yang J, Yang Q, Zhang S, Liu H, Zhou L, et al. Stratified analysis of risk factors affecting vasovagal reactions in first-time whole blood donors: A regional multi-center donor hemovigilance data study. Transfusion Clinique et Biologique. 2024;31(4):237-43.

10. Rahman MM, Karki S, Hayen A. High-frequency whole blood donation and its impact on mortality: Evidence from a data linkage study in Australia. Transfusion. 2024;64(12):2297-305.

11. Lillo-Triguero L, Del Castillo-Rueda A, Bellon JM, Peraita-Adrados R. Prospective study of restless legs syndrome in a blood donors' sample. Rev Clin Esp (Barc). 2025;225(1):9-15.

12. Mukherjee S, Bose A, Sahu A, Mishra D, Prakash S. The impact of isotonic applied muscle tension in reducing the vasovagal event in first-time blood donors: A cohort study. Transfus Clin Biol. 2025;32(2):153-8.

13. Rudokaite J, Ong S, Onal Ertugrul I, Janssen MP, Huis In 't Veld E. Predicting vasovagal reactions to needles from video data using 2D-CNN with GRU and LSTM. PLoS One. 2025;20(1):e0314038.
